# Upper Gastrointestinal Crohn's Disease: Literature Review and Case Presentation

**DOI:** 10.1155/2019/2708909

**Published:** 2019-05-20

**Authors:** Soorya N. Aggarwal, Yana Cavanagh, Lan Wang, Amer Akmal, Matthew A. Grossman

**Affiliations:** ^1^Department of Medicine, Lehigh Valley Health Network, Allentown, Pennsylvania, USA; ^2^Department of Gastroenterology, St. Joseph's University Medical Center, Paterson, New Jersey, USA; ^3^Department of Pathology, St. Joseph's University Medical Center, Paterson, New Jersey, USA

## Abstract

Upper gastrointestinal tract predominant Crohn's Disease (CD) remains an elusive clinical entity, manifesting limited or vague symptomatology, eluding clinical suspicion, and delaying subsequent diagnostic evaluation. As a result, it has not been widely described and there is a lack of clear recommendations for diagnosis or management. Standard IBD evaluation including serologic testing, imaging, and endoscopy may initially not be fruitful. Furthermore, endoscopic evaluation may be grossly normal in patients without long standing-disease. We describe an 18-year-old male who presented with only unexplained, persistent iron-deficiency anemia. Extensive outpatient testing including multiple endoscopic evaluations with standard biopsies was unfruitful. Ultimately, a positive fecal calprotectin prompted enteroscopy with endoscopic mucosal resection (EMR) in an effort to obtain a larger, deeper tissue specimen. Grossly cobblestoned mucosa along with histopathology revealing focal crypt abscesses, chronic inflammation in the lamina propria, and superficial foveolar epithelial regenerative changes were consistent with CD. This patient's case illustrates the need for a high degree of suspicion for CD in patients with unexplained or persistent iron deficiency anemias. Persistent investigation yielded an elevation in fecal calprotectin suggesting underlying gastrointestinal inflammation and prompted advanced endoscopic evaluation with EMR. Waxing and waning tissue findings are characteristic of CD and pose a unique challenge in patients with upper gastrointestinal predominant pathology. As such, diligent workup including laboratory evaluation, imaging, and serial endoscopy is critical to establish pathology and dictate subsequent management in IBD, especially upper gastrointestinal tract predominant CD.

## 1. Introduction

Inflammatory Bowel Disease (IBD) is an umbrella term incorporating ulcerative colitis (UC), Crohn's disease (CD), microscopic colitis, and indeterminate colitis [1–3]. IBD is characterized by cyclic inflammation and healing of the gastrointestinal tract (GIT) and likely results from a complex interplay of genetic predisposition, environmental and psychosocial factors, and dysregulation of gut microbiota [4]. CD can affect any part of the GIT while UC is generally isolated to the colon and rectum. As CD can arise in any part of the GIT, a myriad of clinical presentations may be encountered at diagnosis.

The 2009 American College of Gastroenterology (ACG) management guidelines recommend consideration of CD in patients with unexplained diarrhea, abdominal pain, signs of obstruction, weight loss, fever, or night sweats [5]. When present, these symptoms and physical findings should be corroborated by laboratory abnormalities. Fecal calprotectin had a sensitivity of 0.97 and a specificity of 0.70 in the diagnosis of pediatric IBD in a 2015 meta-analysis [6]. S. cerevisiae antibodies, antineutrophilic cytoplasmic antibodies (ANCA), OmpC (outer-membrane porin C), and genetic tests have also been utilized [5].

Although laboratory findings may be helpful in detecting underlying inflammatory states, they must be corroborated by the clinical picture. The ACG currently describes the standard for diagnosis as a combination of radiographic and endoscopic findings, as well as pathology demonstrating focal, asymmetric, transmural, or granulomatous features [5]. Mucosal disease can be discriminated well with CT enterography but is associated with risk of radiation. As such, MRI has emerged as the most accurate noninvasive tool for assessment of disease extent and distribution [6]. The gold standard, however, for direct visualization of mucosa is endoscopy and is considered a first-line measure for establishing a diagnosis in suspected CD. Endoscopy provides the additional benefit of assessing disease extent and location as well as providing specimens for histopathologic examination [5].

Standard IBD workup including serological testing, imaging, and endoscopic evaluation is most helpful in patients with a high pretest probability of CD. However, lack of classic GI symptomatology or nonspecific symptoms may result in a low clinical suspicion and a subsequent delay in diagnostic testing. A 2013 study published the average time to establish a diagnosis of CD being more than 24 months in 25% of their cohort [7]. Moon et al. attribute the diagnostic delay to a lack of specificity of CD symptoms compounded by a poor accuracy of diagnostic tests [8]. As such, repeat endoscopy is recommended to improve the diagnostic yield and re-examine the GIT, if a diagnosis is not obvious on initial examination [9].

Other diagnostic modalities can include video capsule endoscopy (VCE), which is utilized in detecting small bowel lesions not accessible by standard gastroscopes or colonoscopes [5]. VCE allows for noninvasive, direct visualization of the small bowel mucosa, which can be up to 800 cm long [8, 10]. Unfortunately, capsule retention can occur, particularly in patients with IBD. Cheifetz et al. reported capsule retention in up to 13% of patients with known CD due to the presence of strictures [11]. Atay et al. described retention in 5.2%, in their series of 58 pediatric patients [12]. Push enteroscopy is an endoscopic procedure performed with a longer endoscope, allowing for greater insertion depth and increased mucosal surveillance [13]. Typically, this endoscope can reach the proximal jejunum, or approximately 60 to 120 cm distal to the ligament of Treitz. In one small study, Chong et al. compared VCE and push enteroscopy by evaluating the evidence of intestinal CD provided by each modality. They concluded that VCE visualized small bowel CD more frequently than push enteroscopy [14].

The annual incidence of CD was reported at 20.2 per 100,000/year with a prevalence of 319 per 100,000 in North America [15]. Upper GI predominant CD has not been widely described unlike lower GIT disease. Some reports estimate the prevalence to be between 0.05 and 4%, while others suggest up to 83% of patients with gastrointestinal symptomatology may have isolated upper GI CD [16]. However, lack of specific symptomatology likely results in upper GIT CD remaining undiagnosed until the disease has progressed to involve the lower GIT [17]. Furthermore, clinical and histologic evidence of disease may be discordant. Horje et al. published that 32% of their IBD cohort reported GI symptoms, but only about half of these patients were found to have evidence of IBD on endoscopic evaluation [18]. This demonstrates the notion that symptomatology does not necessarily translate to disease severity or activity [16–18]. Due to this variable correlation between symptoms and pathologic evidence of disease, Kefalas et al. assert that tissue analysis with appropriate sampling can be particularly helpful in the diagnosis of upper GI CD, even in the absence of GI complaints [19]. Annunziata et al. corroborate this finding in a prospective study, reporting that 63% of their cohort with histologic evidence of IBD did not have upper GI complaints [16].

## 2. Case Report

An 18-year-old male initially presented at age 9 with symptomatic iron-deficiency anemia (IDA). He was otherwise healthy and had no family history of GI disorders. Serological evaluation including a leukocyte count, comprehensive metabolic panel, and fecal occult blood testing (FOBT) revealed no abnormalities at that time. Nearly a decade later, persistent IDA in the setting of new FOBT and fecal calprotectin positivity prompted endoscopic evaluation. He was found to have small, sessile polyps in the gastric body and antrum as well as the duodenum with underlying patchy erythema. Tissue biopsy of the gastric mucosa showed moderate, chronic inflammation, without true polyp formation. Biopsy specimens were negative for intraepithelial eosinophils, lymphocytosis, parasites,* H. Pylori*, or intestinal metaplasia. Colonoscopy revealed an ileocecal valve “polyp” that displayed mild, chronic active ileitis not accompanied by villous distortion, intraepithelial lymphocytosis, pyloric metaplasia, or granuloma formation.

**Figure 1:**
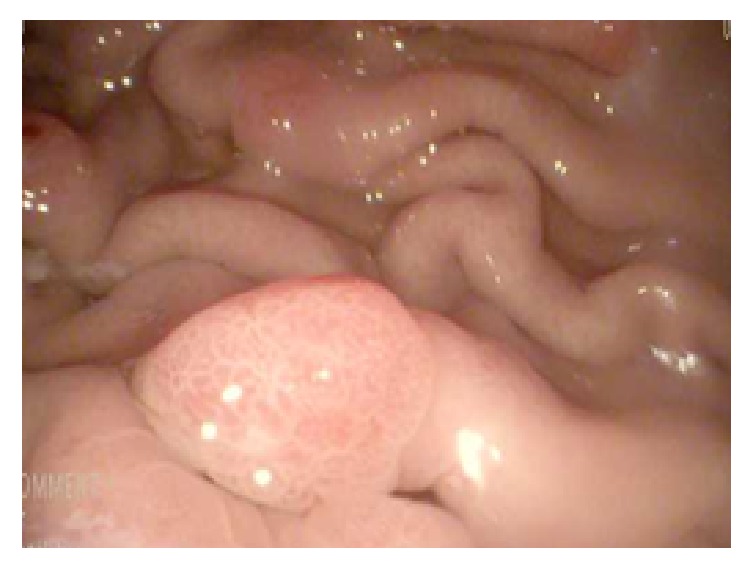
Endoscopic evaluation of the stomach demonstrating polyposis of the mucosa.

**Figure 2:**
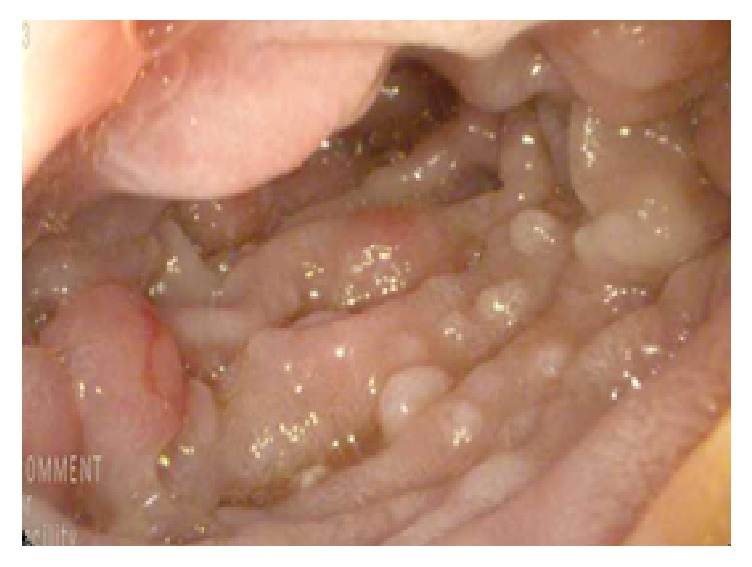
Endoscopic evaluation of the proximal jejunum demonstrating extensive polyposis of the mucosa.

A video capsule endoscopy (VCE) was deployed to evaluate for evidence of small bowel pathology. Multiple small sessile polyps were seen in the stomach; however visualization of the small bowel was limited due to obstruction of visualization by fecal material in the proximal small bowel. VCE was spontaneously passed and a subsequent push enteroscopy was performed to complete examination of the small bowel. Enteroscopy confirmed the presence of numerous polyps, ranging from 4 to 15 mm in size, along the greater curvature of the gastric body (Figure 1), as well as throughout the entire duodenum and in the proximal jejunum (beyond the ligament of Treitz) (Figure 2).

**Figure 3:**
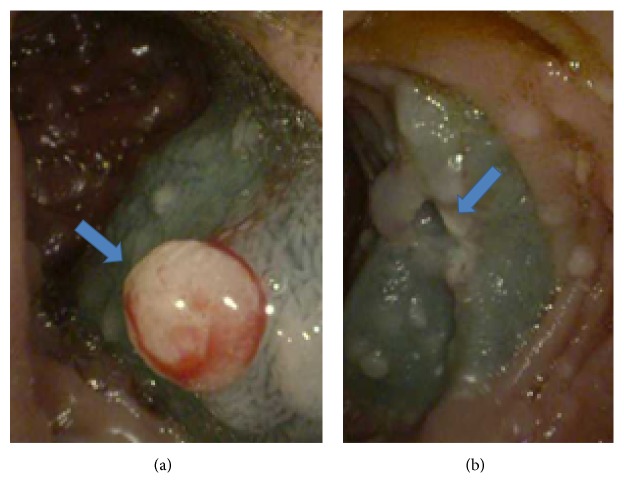
(a) Mucosal lift performed with a solution of methylene blue and saline in preparation for endoscopic mucosal resection of proximal jejunal polypoid lesion (arrow). (b) Mucosal defect post hot snare resection of proximal jejunal polypoid lesion (arrow).

**Figure 4:**
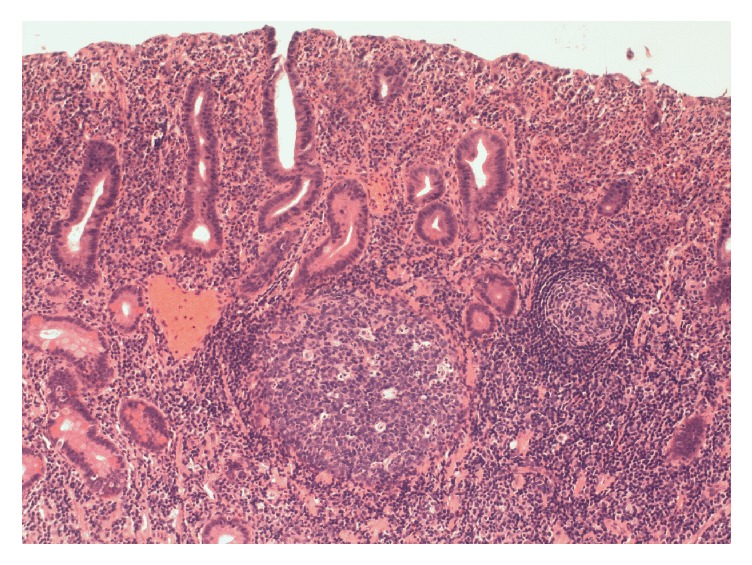
Haematoxylin and eosin (H&E) stain of small bowel tissue sample at low power (100x magnification) demonstrating expansion of the lamina propria by moderately increased chronic inflammation and reactive lymphoid hyperplasia with focal cryptitis, villous blunting, and epithelial regenerative changes consistent with inflammatory pseudopolyps.

**Figure 5:**
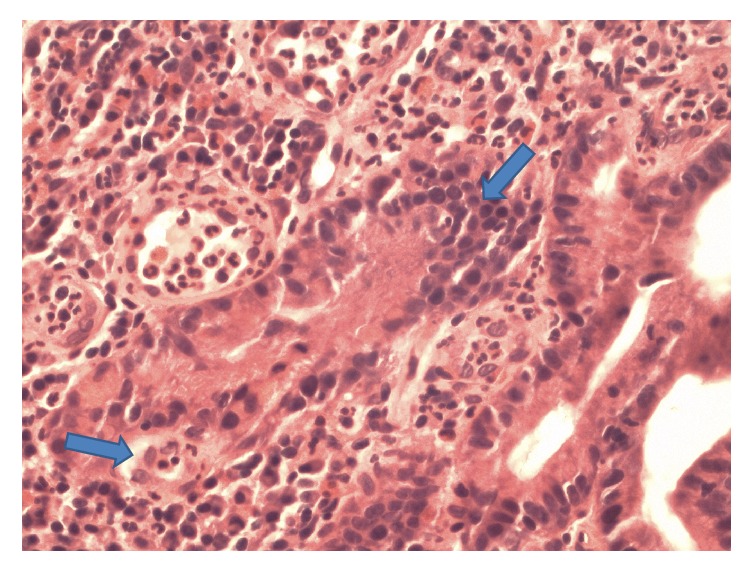
Haematoxylin and eosin (H&E) stain of small bowel tissue sample at high power (400x magnification) demonstrating acute focal cryptitis evidenced by neutrophils within the glandular architecture (arrow), and expansion of the lamina propria by a diffuse neutrophilic infiltrate.

**Figure 6:**
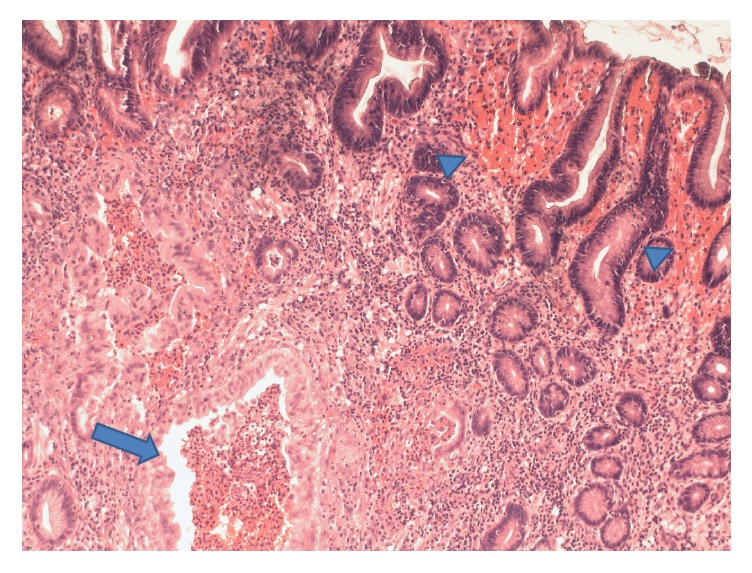
Haematoxylin and eosin (H&E) stain of gastric tissue at low power (100x magnification) demonstrating inflammatory polypoid gastric mucosa, focal crypt abscesses, and increased chronic inflammation in the lamina propria, glandular epithelial reactive changes, and superficial foveolar epithelial regenerative changes.

Biopsies of the polypoid duodenal mucosa and endoscopic mucosal resection (EMR) of the proximal jejunum (Figure 3) revealed focally increased chronic as well as acute inflammation with pseudopolyp formation, evidence of reactive lymphoid hyperplasia in the lamina propria, focal cryptitis, and villous blunting and epithelial regenerative changes (Figures 4 and 5). Sampling of the gastric mucosa revealed inflammatory polypoid gastric mucosa, focal crypt abscesses, and increased chronic inflammation in the lamina propria, glandular epithelial reactive changes, and superficial foveolar epithelial regenerative changes (Figure 6). No increased intraepithelial lymphocytosis, granuloma, or dysplasia was identified.

## 3. Discussion

We encountered a young patient without gastrointestinal complaints for evaluation of unexplained, persistent anemia. Anemia in a male patient with no alternate etiologies of blood loss generally warrants further evaluation and consideration of underlying celiac disease, IBD or malignancy. A 2014 meta-analysis of the prevalence of anemia in IBD patients cited that up to 27% of all patients with CD had clinically significant anemia. When considering the pediatric population, Gerasimidis et al. published that up to 72% of their pediatric cohort was anemic at the time of diagnosis [20]. In this patient, the inflammatory etiology of his pathology was likely contributing to the ongoing anemia. However, lack of characteristic histopathologic findings prior to enteroscopy with EMR made it difficult to establish a diagnosis.

Cobblestoning is a result of submucosal edema while inflammatory polyps are the result of overcompensated healing of inflamed and damaged mucosa. A cobblestoned appearance refers to the gross mucosal pattern of longitudinal ulcers or fissures separating islands of mucosa and sometimes containing pseudopolyps [21, 22]. Inflammatory polyps consist of granulation tissue with a mixture of lymphocytes, plasma cells, mast cells, neutrophils, and eosinophils. Based on the stage of inflammation, they can have varying degrees of re-epithelialization and varying amounts of granulation tissue, reflecting the stages of healing [2]. Foveolar reactive change, found in the gastric mucosa of our patient, is a type of reactive gastritis that was reported to be essential for the formation of inflammatory polyps by Mitsufuji et al. [23, 24].

Although the diagnosis of CD has typically relied on the identification of granulomas, varying inflammatory pathology is emerging as suggestive or diagnostic of IBD [16]. Ruska et al. described endoscopic findings in a pediatric IBD cohort ranging from esophagitis (16 patients), esophageal ulcers (2 patients), nonspecific gastritis (22 patients), duodenitis, and duodenal ulcers (18 patients) [25]. This variance of histologic presentation suggests that CD may present atypically on endoscopy and direct tissue examination, particularly when involving the upper GIT. In their literature review, Wright et al. even categorized a pattern of focal, acute,* H. Pylori* negative gastritis, and duodenitis as a newly described presentation of CD [16]. In fact, multiple authors suggest that encountering* H. Pylori* negative duodenitis or gastritis in the absence of chronic NSAID use is highly suggestive of underlying IBD in patients without previously documented IBD for whom CD of the upper GIT is suspected [5, 17, 26].

Notably, EMR may have assisted in diagnosing isolated upper GI CD in this patient. Biopsies obtained during initial upper endoscopy yielded incomplete submucosa and failed to provide compelling histologic evidence for IBD. In contrast, EMR provided preserved tissue architecture that was consistent with a histological diagnosis of upper GI CD. According to American Society for Gastrointestinal Endoscopy, EMR may be helpful in obtaining histologic diagnoses from the mucosa as well as subepithelial lesions located in the muscularis mucosa or in the superficial submucosa [24]. Furthermore, EMR may be implemented when standard sampling techniques, such as jumbo biopsy forceps, fail to provide adequate tissue specimen.

Upper GI predominant CD is a rare and diagnostically challenging presentation of IBD. It has yet to be described in detail in the medical literature. This may be due to a lack of specific clinical symptoms and, thus, heavier reliance on tissue diagnosis, as well as the limited number of records describing new onset CD isolated to the upper GI tract. Available data therefore underestimates the true prevalence of upper GI CD. As such, it is pivotal for clinicians to understand how these nuanced these patient presentations may present. In our patient, establishing the diagnosis of CD was a challenge due to a lack of gastrointestinal symptoms and non-classical pathologic findings. However, the combination of IDA, elevated fecal calprotectin, and inflammatory polyposis with evidence of focal, chronic inflammation in the setting of a young male was all highly suggestive of upper GI CD.

## Additional Points

*Core Tip*. Identification of upper gastrointestinal predominant Crohn's Disease is an uncommon initial diagnosis due to lack of specific symptoms. Despite this, early diagnosis of Crohn's Disease is important to initiating therapy and allaying progression of disease. Understanding clinical presentation and serology as well as histopathologic features including inflammatory polyps or pseudopolyp formation in Crohn's Disease is crucial in the timely diagnosis of IBD.
